# Dietary Protein Intake, Protein Energy Wasting, and the Progression of Chronic Kidney Disease: Analysis from the KNOW-CKD Study

**DOI:** 10.3390/nu11010121

**Published:** 2019-01-08

**Authors:** Sung Woo Lee, Yong-Soo Kim, Yeong Hoon Kim, Wookyung Chung, Sue K. Park, Kyu Hun Choi, Curie Ahn, Kook-Hwan Oh

**Affiliations:** 1 Department of Internal Medicine, Nowon Eulji Medical Center, Eulji University, Seoul 01830, Korea; neplsw@gmail.com; 2 Department of Internal Medicine, College of Medicine, Catholic University of Korea, Seoul 06591, Korea; kimcmc@catholic.ac.kr; 3 Department of Internal Medicine, Inje University, Busan Paik Hospital, Busan 47392, Korea; yeonghnl@inje.ac.kr; 4 Department of Internal Medicine, Gachon University, Gil Hospital, Incheon 21565, Korea; jwkpsj79@hanmail.net; 5 Department of Preventive Medicine, Seoul National University College of Medicine, Seoul 03082, Korea; suepark@snu.ac.kr; 6 Cancer Research Institute, Seoul National University, Seoul 08826, Korea; 7 Department of Biomedical Science, Seoul National University Graduate School, Seoul 03081, Korea; 8 Department of Internal Medicine, College of Medicine, Institute of Kidney Disease Research, Yonsei University, Seoul 03722, Korea; khchoi6@yumc.yonsei.ac.kr; 9 Department of Internal Medicine, Seoul National University Hospital, Seoul 03080, Korea; curie@snu.ac.kr

**Keywords:** dietary protein intake, protein energy wasting, chronic kidney disease, progression, nutrition

## Abstract

Studies on the effect of dietary protein intake (DPI) on chronic kidney disease (CKD) progression, along with the potential hazard of protein-energy wasting (PEW), are scarce. We evaluated the association between DPI and kidney function both cross-sectionally and longitudinally, particularly emphasizing the role of PEW, in a large-scale, observational, multicenter, prospective study. We enrolled 1572 patients with non-dialysis CKD between 2011 and 2016. CKD progression was defined by a >50% estimated glomerular filtration rate (eGFR) decrease, serum creatinine doubling, or dialysis initiation. A Cox proportional hazard regression analysis was conducted. During the mean follow-up period of 41.6 months, CKD progression was observed in 296 patients. Cross-sectionally, increased DPI was significantly associated with increased eGFR. Similarly, increased DPI tertile was significantly associated with increased renal survival in a Kaplan–Meier curve analysis. In the multivariate Cox proportional hazard regression analysis, the statistical significance of the DPI tertile group in CKD progression was lost when PEW-related variables were added as covariates. In penalized spline curve analysis, the adjusted odds ratio of PEW significantly increased as DPI decreased. DPI, per se was not a major determinant of CKD progression. An intimate association between reduced DPI and PEW may be a more important predictor of CKD progression than DPI.

## 1. Introduction

Since most uremic toxins are waste products of protein metabolism [1], a low-protein diet (LPD) may be effective in alleviating uremic symptoms in patients with advanced chronic kidney disease (CKD) [2,3]. Because early dialysis initiation did not show survival benefits in patients with CKD [4], LPD as a conservative approach to delay dialysis initiation has been revisited [5,6]. An LPD not only simply delays dialysis initiation but also may slow CKD progression by the reduction of renal hyperfiltration [7,8,9]. Nonetheless, LPDs have not been frequently used [10] because the cornerstone trial, the Modification of Diet in Renal Disease (MDRD) study, failed to demonstrate a clear benefit of LPDs on slowing CKD progression [11], and long-term follow-up of the MDRD study has warned that LPDs may be a potential hazard to patients’ survival [12].

Patients with CKD are exposed to a high risk of developing syndromes of muscle wasting, malnutrition, and inflammation. Recently, the term protein energy wasting (PEW) was suggested to describe this syndromic uremic malnutrition [13]. As CKD progresses, the risk of PEW is increased [14], which ultimately results in increased risk of mortality [15]. Patients with CKD are easily malnourished with an LPD [16], as participants who consumed an LPD in the MDRD study were [17]. Nonetheless, subsequent studies after the MDRD study have shown renoprotective effects of LPDs in patients with CKD [18,19], particularly when consumption of an LPD was well conducted with good nutritional supplements [20]. Therefore, the use of an LPD is still a promising treatment option to delay CKD progression if clinicians are careful of the hazard of PEW. In this regard, an LPD, a reduced dietary protein intake (DPI) without PEW, should be separated from DPI per se.

Unintentionally low DPI is a key component of PEW [13], suggesting reduced DPI and PEW are inseparable. Therefore, the effect of DPI per se on CKD progression should be analyzed in conjunction with the potential hazard of PEW on kidney health. However, no studies have incorporated the concept of PEW when evaluating the association between DPI and kidney function. In addition, the association between DPI per se and kidney function remains unclear [21,22,23,24,25]. Moreover, most of the previous studies have used data from the general population [21,22,23,24], not patients with CKD, which may attenuate the potential effects of PEW. This study aimed to evaluate the association between DPI and kidney function both cross-sectionally and longitudinally, particularly emphasizing the role of PEW, using data from a large number of adults who were enrolled in the KoreaN cohort study for Outcome in patients With Chronic Kidney Disease (KNOW-CKD).

## 2. Materials and Methods

### 2.1. Participants

The KNOW-CKD is a multicenter prospective cohort study in Korea that enrolled 2238 patients with non-dialysis CKD stages 1–5 between February 2011 and January 2016. The detailed design and methods of the KNOW-CKD were published previously (NCT01630486 at http://www.clinicaltrials.gov) [26]. The protocol of the KNOW-CKD adhered to the principles of the Declaration of Helsinki and was approved by the Institutional Review Board at each participating hospital, including the Seoul National University Hospital, Yonsei University Severance Hospital, Kangbuk Samsung Medical Center, Seoul St. Mary’s Hospital, Gil Hospital, Eulji Medical Center, Chonnam National University Hospital, and Pusan Paik Hospital. Written informed consent was obtained from all subjects. Patients were clinically followed according to the policy of participating hospitals, and blood and urine were tested and stored annually from the date of enrollment. The estimated glomerular filtration rate (eGFR) was calculated using a Chronic Kidney Disease Epidemiology Collaboration equation [27]. CKD and the stages were defined using the Kidney Disease Improving Global Outcomes 2012 guidelines [28].

Of the 2238 patients, 319 were excluded; these included 247 with missing 24-h urine creatinine (Cr), 55 with missing 24-h urine urea, 3 with missing data to calculate the Cr index (CRI), 10 with missing serum albumin, and 4 with missing cholesterol. Of the 1919 eligible patients, 347 were further excluded because of incomplete 24-h urine collection. Therefore, 1572 patients were included in the final analysis.

### 2.2. Measurement

Clinical data, including detailed demographic information and baseline laboratory results, were extracted from the electronic data management system (PhactaX). Body mass index (BMI) was calculated as weight (kg) per square of height (m^2^). Serum Cr levels were measured at a central laboratory (Lab Genomics, Seongnam, Korea). Completeness of 24-h urine collection was ascertained by CRI ≥ 0.7 [29]. CRI = measured 24-h urine Cr/expected 24-h urine Cr, where the expected 24-h urine Cr was calculated by the Tanaka equation: −2.04 × age + 14.89 × weight (kg) + 16.14 × height (cm) − 2244.45 [30]. Daily protein intake was assessed using 24-h urinary urea nitrogen (UUN): estimated daily protein intake (g/day) = 6.25 × (24-h UUN (g/day) + 0.031 (g/kg/day) × ideal body weight (kg)) [31], where ideal body weight was calculated assuming BMI 22.5 kg/m^2^ as the optimal value [32]. DPI was expressed as the normalized estimated daily protein intake divided by ideal body weight (g/kg/day). Estimated skeletal muscle mass (SMM) was calculated using 24-h urine Cr excretion: SMM (kg) = 18.9 × measured 24-h urine Cr (mg/day) × 0.001 + 4.1 [33].

### 2.3. Definitions

Hypertension was determined by physician diagnosis, systolic blood pressure (BP) ≥ 140 mm Hg or diastolic BP ≥ 90 mmHg, or treatment with antihypertensive drugs. Diabetes was determined by physician diagnosis, fasting glucose ≥ 126 mg/dL, or treatment with insulin or oral antidiabetic drugs. Alcohol drinking was defined as drinking alcoholic beverages more than once a month. PEW components were created based on the International Society of Renal Nutrition and Metabolism diagnostic criteria [13]. An expert panel recommended that four main categories be recognized for the diagnosis of PEW: biochemical indicators, low body mass, decreased muscle mass, and low protein intake. Since protein intake was our main factor, two biochemical indicators (serum albumin and cholesterol), one low BMI measure, and decreased muscle mass (estimated SMM) were used instead to define PEW. Therefore, the four PEW components were low serum albumin, low cholesterol, low BMI, and low estimated SMM, which were defined as the lowest quartiles of the respective variables: serum albumin < 40.0 g/L, cholesterol < 3.8 mmol/L, BMI < 22.3 kg/m^2^, and sex-specific estimated SMM < 19.7 kg in women and <26.9 kg in men. Three or more of the four PEW components had to be present for the diagnosis of PEW. Advanced CKD was defined as eGFR < 45 mL/min/1.73 m^2^ (stage 3b/4/5).

Tertiles of DPI were <0.93 g/kg/day in the first tertile, 0.93–1.16 g/kg/day in the second tertile, and ≥1.16 g/kg/day in the third tertile, respectively. The main outcome was the development of renal events, which were defined by a decrease in eGFR of >50% from the baseline values, doubling of serum Cr, or dialysis initiation, and these were detected annually.

### 2.4. Missing Values

The following were the variables with missing values: current smoking (1/1572, 0.1%), alcohol drinking (113/1572, 7.2%), hypertension (1/1572, 0.1%), diabetes (5/1572, 0.3%), causes of CKD (1/1572, 0.1%), bilirubin (1/1572, 0.1%), fasting plasma glucose (2/1572, 0.1%), hemoglobin (9/1572, 0.6%), urine protein-to-Cr ratio (UPCR, 38/1572, 2.4%), and high sensitivity C-reactive protein (hsCRP, 73/1572, 4.6%).

### 2.5. Statistical Analysis

The distributions of continuous variables were evaluated using histograms and Q-Q plots. Two variables, hsCRP and UPCR, were not normally distributed. Normally distributed continuous variables are expressed as mean ± standard deviation, non-normally distributed continuous variables as median (interquartile range), and categorical variables as percentages. *p*-trends were analyzed for normally distributed continuous variables using a linear-term of the one-way analysis of variance (ANOVA); for non-normally distributed continuous variables, Jonckheere–Terpstra tests were used; and for categorical variables, a linear-by-linear association was used. Differences were analyzed using a Bonferroni post hoc analysis of one-way ANOVA for normally distributed continuous variables, Mann–Whitney U tests for non-normally distributed continuous variables, and chi-square tests for categorical variables. Death before renal events was treated as a censored observation. For the survival analysis, the Kaplan–Meier curve was employed, and the statistical significance was calculated using the log-rank test. The hazard ratio (HR) and its 95% confidence interval (CI) for renal events was assessed using the Cox proportional hazard regression analysis. The assumption of proportional hazard was tested by log minus log plot for categorical variables and interaction analysis with time covariate using time-dependent Cox regression for continuous variables. Since DPI, blood urea nitrogen (BUN), eGFR, bilirubin, serum albumin, and hemoglobin did not meet the proportion hazard assumption, categorical versions of these variables (tertile of DPI, eGFR < 45 mL/min/1.73 m^2^, and lowest quartile values of BUN < 6.0 mmol/L, bilirubin < 8.6 μmol/L, hemoglobin < 11.5 g/dL, and serum albumin < 40.0 g/L) were used instead for the Cox proportional hazard regression analysis. Odds ratios (OR) and 95% CI were calculated using the logistic regression analysis. In multivariate analyses, covariates were chosen based on their clinical and statistical relevance: age, sex, current smoking, alcohol drinking, hypertension, diabetes, causes of CKD, UPCR, BUN, eGFR, bilirubin, hemoglobin, and hsCRP. The multivariate generalized additive model (GAM) for Gaussian distribution [34] was adapted to visualize the associations between DPI and eGFR using the “mgcv” package in R Statistics (version 3.03, R Foundation for Statistical Computing, Vienna, Austria). The relationship between DPI and PEW was plotted with the penalized smoothing spline method, using the “pspline” package in R statistics (version 3.03). A *p* value of < 0.05 was considered statistically significant. All analyses, unless otherwise specified, were performed using SPSS version 22 (IBM Corp., released 2013, Armonk, NY, USA).

## 3. Results

The mean age of the 1572 patients was 54.3 years, and 63.1% were men. The causes of CKD were diabetic nephropathy (23.0%), hypertensive nephropathy (20.7%), glomerulonephritis (32.9%), and other causes (23.3%). The mean eGFR was 54.6 mL/min/1.73 m^2^, and the median UPCR was 0.47 g/g Cr. The stages of CKD were 17.0% in stage 1, 20.2% in stage 2, 16.3% in stage 3a, 21.5% in stage 3b, 19.8% in stage 4, and 5.2% in stage 5. The mean DPI of the patients was 1.1 g/kg/day. The mean values of BMI, estimated SMM, serum albumin, and cholesterol were 24.5 kg/m^2^, 28.3 kg, 42.1 g/L, and 4.5 mmol/L, respectively. The overall prevalence of PEW was 6.8%. During the mean follow-up period of 41.6 months, 296 patients (18.8%) experienced renal events, 172 patients (10.9%) had >50% eGFR decline or serum Cr doubling, and 235 patients (14.9%) had dialysis initiation (Figure S1). The percentages of renal events/≥50% eGFR decline or serum Cr doubling/dialysis initiation were 0.4%/0.4%/0.0% in stage 1, 4.1%/4.1%/1.6% in stage 2, 8.6%/7.0%/4.3% in stage 3a, 17.8%/16.6%/11.5% in stage 3b, 43.3%/22.4%/37.2% in stage 4, and 79.3%/17.1%/78.0% in stage 5.

The baseline characteristics of the DPI tertile group were compared (Table 1). As the DPI tertile increased, the proportion of males and the alcohol drinking rate significantly increased. However, no difference was observed in patient age, current smoking rate, rates of hypertension and diabetes, and causes of CKD between the DPI tertile groups. With the increase of DPI tertile, eGFR and UPCR increased and decreased, respectively, leading to a decreased percentage of advanced CKD. Although BUN levels were not affected by the DPI tertile, BMI, serum albumin, and estimated SMM significantly increased as the DPI tertile increased. As the DPI tertile increased, hemoglobin levels significantly increased.

**Figure 1:**
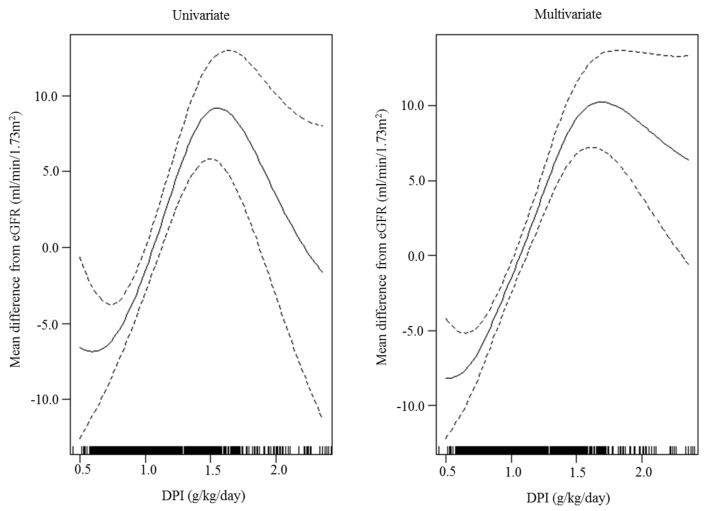
Generalized additive model (GAM) plot for Gaussian distribution between dietary protein intake (DPI) and estimated glomerular filtration rate (eGFR). The solid and dashed lines are the spline curve and its 95% confidence intervals, respectively. The upper (≥2.36 g/kg/day) and lower 1% (<0.5 g/kg/day) of DPI were truncated. The covariates in the multivariate GAM analysis were age, sex, current smoking, alcohol drinking, hypertension, diabetes, causes of chronic kidney disease, urine protein to creatinine ratio, blood urea nitrogen, bilirubin, hemoglobin, body mass index, cholesterol, estimated skeletal muscle mass, and serum albumin.

**Figure 2:**
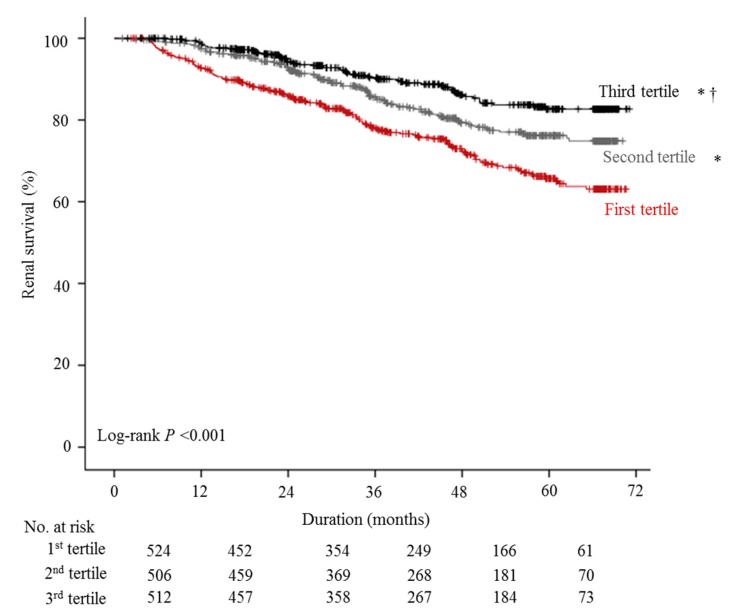
Kaplan–Meier survival curve of tertiles of dietary protein intake (DPI). * and **^†^** indicate *p* < 0.05 when compared to the first and second tertiles of the DPI group, respectively, using the log-rank test.

The association between DPI and eGFR was analyzed cross-sectionally using the multivariate GAM plot, which revealed that increased DPI was significantly associated with an increased eGFR (Figure 1). In the longitudinal assessment (Figure 2), the estimated renal survival using the Kaplan–Meier method was significantly increased with an increase in DPI tertile; the mean values (95% CI) of the estimated renal survivals were 56.4 (54.4–58.4) months in the first tertile, 60.9 (59.2–62.6) months in the second tertile, and 64.6 (63.1–66.1) months in the third tertile (Log-rank *p* < 0.001). This was confirmed in multivariate Cox proportional hazard regression analysis (Table 2, Table S1) by entering age, sex, smoking and drinking status, hypertension, diabetes, causes of CKD, UPCR, BUN, eGFR, bilirubin, hemoglobin, and hsCRP as covariates. The HR (95% CI) of the third vs. first tertile was 0.685 (0.495–0.948, *p* = 0.02). However, the statistical significance of the DPI tertile group on renal event development was lost when variables related to PEW were added as covariates (Model 2). In the sensitivity analysis according to the various PEW conditions (Table 3), DPI tertile lost statistical significance as the statistical significance of various PEW conditions became evident (Models 3–6), although interactions between various PEW conditions and the DPI tertile group for renal event development were not statistically significant.

**Figure 3:**
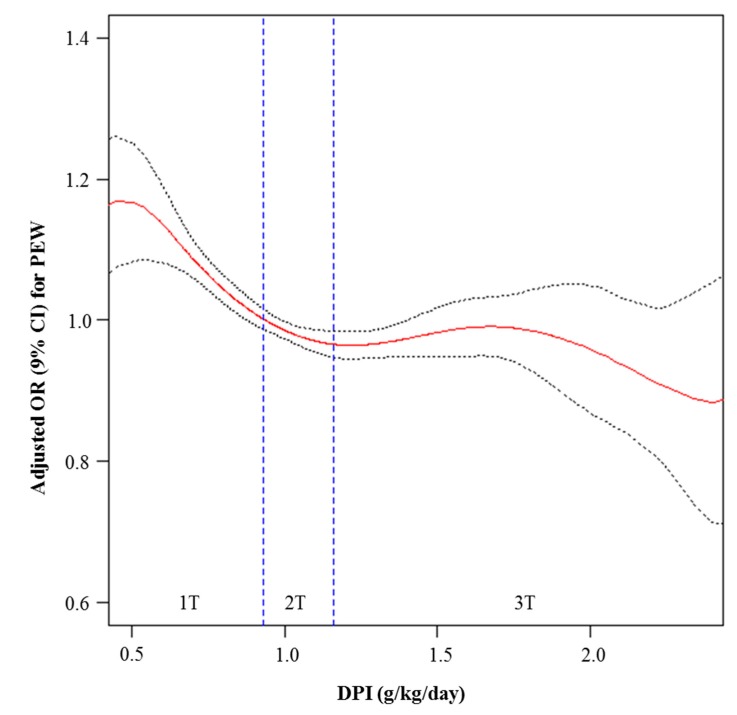
Penalized smoothing splines showing the relationship between DPI and PEW. DPI, dietary protein intake; PEW, protein energy wasting; OR, odds ratio; CI, confidence interval; T, tertile. The upper (≥2.36 g/kg/day) and lower 1% (<0.5 g/kg/day) of DPI were truncated. The red line indicates the OR and the black dotted line indicates the 95% CI for which DPI influences PEW components by three or more. OR and 95% CI were analyzed using a multivariate logistic regression analysis including age, sex, current smoking, alcohol drinking, hypertension, diabetes, causes of chronic kidney disease, urine protein to creatinine ratio, blood urea nitrogen, estimated glomerular filtration rate, bilirubin, hemoglobin, and high sensitivity C-reactive protein.

The association between DPI and PEW was assessed. In the penalized spline curve analysis using multivariate logistic regression analysis, the adjusted OR for PEW significantly increased as DPI decreased, particularly in the first tertile of DPI (Figure 3). We also analyzed the association between DPI and the respective PEW components. In the multivariate analysis, the odds for a low BMI and estimated SMM significantly decreased when DPI increased, whereas DPI was not associated with low serum albumin and cholesterol (Supplemental Table S2).

## 4. Discussion

In this study, we evaluated the association between DPI and kidney function both cross-sectionally and longitudinally and found that DPI per se was not a major determinant of CKD progression; instead, an intimate association between reduced DPI and PEW may be a more important predictor of CKD progression.

Ever since the MDRD study failed to prove the benefits of an LPD to postpone CKD progression [11], the use of an LPD as an important treatment option in patients with CKD has been doubtful. Nonetheless, numerous studies have been conducted to compensate the failure of the MDRD study because consumption of an LPD is hypothetically excellent [35]. In this regard, a recent study by Garneata et al., successfully revisited the clinical usefulness of LPD consumption to delay CKD progression [20]. To overcome the low compliance issue, they adopted a vegetarian LPD, instead of the traditional LPD [36]. In addition, they supplied a ketoanalogue to prevent potential LPD-related malnutrition. Unlike the MDRD study, the study by Garneata et al., successfully proved the beneficial effects of LPD consumption on CKD progression without significant malnutrition. These two important antithetic study results suggest that LPD consumption is a very effective treatment option, if the diet is well-organized and prepared. However, this may be too ideal. Not all clinics can provide a good LPD to patients with CKD. Therefore, the results from well-organized LPDs should not be extrapolated as the effect of DPI per se on CKD progression, because the lower the DPI is, the higher the PEW that can be developed is [14,36]. Although several studies have reported the relationship between DPI per se and kidney function [21,22,23,24,25,37,38,39], no studies evaluating the effect of DPI on CKD progression in conjunction with the potential hazard of PEW have been conducted.

In this study, we identified cross-sectionally using a multivariate GAM plot that increased DPI was significantly associated with increased eGFR. This is in accordance with the original hypothesis by Brenner that a high-protein intake induces renal hyperfiltration [8], which was confirmed in a recent population-based study [40]. Generally, this positive association between DPI and eGFR has been thought to be harmful on renal survival, and as Cirillo et al. reported [21], increased DPI was expected to be associated with an increased risk of CKD progression. Unlike Cirillo et al., however, we found that increased DPI was significantly associated with a decreased risk of development of renal event development, which was independent from demographic and comorbid confounders. To identify the reason of benefit of increased DPI on renal survival, the model with various PEW conditions was further adjusted, which ultimately nullified the association between DPI and development of renal events. This ultimate null association between DPI and CKD progression was quite similar to the results of Metzger et al. [25]. In their uncorrected DPI results, increased DPI was associated with a decreased risk of development of renal events (HR 0.92, 95% CI 0.86–0.99) in a univariate analysis. However, DPI per se was not associated with renal events in a multivariate analysis using BMI and serum albumin as covariates (HR 1.06, 95% CI 0.99–1.14). Based on these results, we conclude that modifying DPI per se may have no impact on renal outcomes, unless associated PEW is well-controlled.

To prove that the decreased DPI in this study was not part of well-organized LPD, we further analyzed the association between DPI and PEW and found that decreased DPI was significantly associated with increased odds for PEW. In addition, we identified that increased DPI was independently associated with increased odds of body masses (BMI and SMM), whereas DPI was not directly associated with serum albumin and cholesterol. These results suggest several clinical meanings. First, in an ordinary outpatient clinic, decreased DPI may not be a sign of good LPD; rather it may be a red flag of the beginning of PEW in patients with CKD, which finally can deteriorate kidney function. Second, to build up patients’ body mass, increased DPI may be a good treatment option without fear of CKD deterioration, at least in Korean patients with CKD. Finally, increased DPI only may not be effective to increase serum levels of albumin and cholesterol, suggesting a much more complex pathogenesis in the derangement of biochemical parameters.

This study has several limitations. First, the choice and definition of PEW components in this study did not strictly follow the International Society of Renal Nutrition and Metabolism diagnostic criteria [13]. However, the current study provided evidence that our working definition of PEW was valid at least in predicting CKD progression. Moreover, previous studies also did not strictly follow the recommendations because of the uncertainty in the diagnostic criteria [15,41,42]. Second, other nutritional data including daily calorie intake and body composition data including fat distribution and lean mass were not available because KNOW-CKD was not originally designed to identify the effect of DPI and PEW on renal outcomes. We also had no data on whether patients were actively on LPD programs and well-informed about the hazard of PEW. Therefore, it is not clear how many patients were on well-organized LPD programs. However, the intimate relationship between reduced DPI and PEW may suggest that LPD programs, if any, were inappropriate. Third, potential benefits of a reduced DPI could not be shown because of the strong association between reduced DPI and PEW. Finally, the use of a single nation and ethnicity limits the generalizability. In this study, the mean DPI of Korean patients with CKD was only 1.1 g/kg/day, which is far lower than that of US patients with CKD (1.3 g/kg/day) [43], suggesting that the potential U-shaped association between DPI and renal outcomes [24] could have been masked because of the relatively low DPI in Korean patients with CKD.

In conclusion, DPI per se was not shown to be a major determinant of future development of renal events. Instead, PEW was associated more with CKD progression. Therefore, clinicians should cautiously interpret the meaning of reduced DPI because this can be the red flag of PEW, particularly when patients are not on well-organized LPD programs. Future studies are necessary to confirm our study results.

## Figures and Tables

**Table 1:** Baseline characteristics of patients in the DPI tertile group.

	Tertile of DPI			*p*-trend
	1T (*n* = 533)	2T (*n* = 512)	3T (*n* = 527)	
Age (years)	54.1 ± 12.0	54.0 ± 12.1	54.7 ± 12.0	0.35
Male sex (%)	58.9	61.3	69.1 *^,†^	0.001
Current smoking (%)	15.6	16.8	14.1	0.50
Alcohol drinking (%)	39.9	43.7	53.7 *^,†^	<0.001
Hypertension (%)	94.9	94.9	95.6	0.60
Diabetes (%)	24.7	23.7	25.5	0.78
Cause of CKD				
DMN (%)	25.0	23.0	21.1	0.14
HN (%)	18.6	21.9	21.9	0.19
GN (%)	34.1	32.4	32.1	0.48
Others (%)	22.3	22.7	24.9	0.32
BUN (mmol/L)	9.9 ± 5.9	9.9 ± 5.3	9.5 ± 4.9	0.19
Cr (μmol/L)	175.5 ± 109.7	154.2 ± 86.3 *	132.9 ± 67.4 *^,†^	<0.001
eGFR (ml/min/1.73 m^2^)	49.2 ± 30.9	53.9 ± 30.5 *	60.8 ± 29.3 *^,†^	<0.001
UPCR (g/g Cr)	0.54 (0.17–1.63)	0.45 (0.12–1.40) *	0.40 (0.12–1.03) *	0.001
Advanced CKD (%)	55.3	47.7 *	36.6 *^,†^	<0.001
DPI (g/kg/day)	0.78 ± 0.12	1.04 ± 0.07 *	1.48 ± 0.41 *^,†^	<0.001
Bilirubin (μmol/L)	11.1 ± 4.8	11.9 ± 5.1 *	12.3 ± 5.3 *	<0.001
BMI (kg/m^2^)	23.4 ± 3.2	24.6 ± 3.2 *	25.4 ± 3.1 *^,†^	<0.001
FPG (mmol/L)	5.9 ± 1.8	6.1 ± 2.2	6.1 ± 1.9	0.17
Serum albumin (g/L)	41.6 ± 4.5	42.3 ± 3.8 *	42.5 ± 3.7 *	<0.001
Cholesterol (mmol/L)	4.4 ± 1.1	4.5 ± 1.0	4.5 ± 0.9	0.27
Estimated SMM (kg)	24.8 ± 5.6	28.0 ± 6.2 *	32.0 ± 7.8 *^,†^	<0.001
Hemoglobin (g/dL)	12.4 ± 2.0	13.0 ± 2.0 *	13.5 ± 1.9 *^,†^	<0.001
hsCRP (nmol/L)	5.7 (1.9–16.2)	5.3 (1.9–14.1)	6.7 (2.4–16.2)	0.39

T, tertile; CKD, chronic kidney disease; DMN, diabetic nephropathy; HN, hypertensive nephropathy; GN, glomerulonephritis; BUN, blood urea nitrogen; Cr, creatinine; eGFR, estimated glomerular filtration rate; UPCR, urine protein-to-creatinine ratio; DPI, dietary protein intake; BMI, body mass index; FPG, fasting plasma glucose; SMM, skeletal muscle mass; hsCRP, high sensitivity C-reactive protein. Values are expressed as mean ± standard deviation for normally distributed continuous variables, median (interquartile range) for non-normally distributed continuous variables, and percentage for categorical variables. *p*-trends were analyzed by the linear term of the one-way ANOVA for normally distributed continuous variables, the Jonckheere–Terpstra test for non-normally distributed continuous variables, and a linear-by-linear association for categorical variables. Tertiles of DPI were <0.93 g/kg/day in the first tertile, 0.93–1.16 g/kg/day in the second tertile, and ≥1.16 g/kg/day in the third tertile, respectively. * and **^†^** indicate *p* < 0.05 when compared to 1T and 2T of the DPI group, respectively, using Bonferroni post-hoc analysis of one-way ANOVA for normally distributed continuous variables, the Mann–Whitney U test for non-normally distributed continuous variables w, and the chi-square test for categorical variables.

**Table 2:** Effect of DPI on renal survival.

	Univariate		Model 1		Model 2	
	HR (95% CI)	*p*	HR (95% CI)	*p*	HR (95% CI)	*p*
DPI tertile						
Second tertile vs. first tertile	0.626 (0.482–0.814)	<0.001	0.765 (0.576–1.016)	0.07	0.810 (0.600–1.094)	0.17
Third tertile vs. first tertile	0.412 (0.306–0.556)	<0.001	0.685 (0.495–0.948)	0.02	0.737 (0.516–1.054)	0.10
BMI (kg/m^2^)	-	-	-	-	1.013 (0.966–1.062)	0.59
Estimated SMM (kg)	-	-	-	-	0.989 (0.961–1.019)	0.48
Cholesterol < 3.8 mmol/L (yes vs. no)	-	-	-	-	1.030 (0.777–1.364)	0.84
Serum albumin < 40.0 g/L (yes vs. no)	-	-	-	-	1.438 (1.051–1.969)	0.02

DPI, dietary protein intake; HR, hazard ratio; CI, confidence interval; BMI, body mass index; SMM, skeletal muscle mass. HR and 95% CI were analyzed using Cox proportional hazard regression analysis. In the multivariate analysis, the covariates in model 1 were age, sex, current smoking, alcohol drinking, hypertension, diabetes, causes of chronic kidney disease, urine protein to creatinine ratio, blood urea nitrogen, estimated glomerular filtration rate, bilirubin, hemoglobin, and high sensitivity C-reactive protein. The covariates in model 2 were variables in model 1 with BMI, estimated SMM, cholesterol, and serum albumin.

**Table 3:** Sensitivity analysis based on different protein-energy wasting (PEW) conditions.

	Model 3		Model 4		Model 5		Model 6	
	HR (95% CI)	*p*	HR (95% CI)	*p*	HR (95% CI)	*p*	HR (95% CI)	*p*
DPI tertile (vs. first tertile)								
Second tertile	0.812 (0.603–1.092)	0.17	0.763 (0.572–1.018)	0.07	0.792 (0.591–1.063)	0.12	0.828 (0.620–1.105)	0.20
Third tertile	0.714 (0.511–0.998)	0.05	0.684 (0.491–0.952)	0.02	0.705 (0.506–0.983)	0.04	0.725 (0.523–1.006)	0.05
No. of PEW components (vs. 0)								
1	1.029 (0.745–1.420)	0.86	-	-	-	-	-	-
2	0.913 (0.619–1.346)	0.64	-	-	-	-	-	-
≥3	1.800 (1.181–2.742)	0.01	-	-	-	-	-	-
PEW components ≥1 (yes vs. no)	-	-	1.077 (0.799–1.452)	0.63	-	-	-	-
PEW components ≥2 (yes vs. no)	-	-	-	-	1.131 (0.860–1.487)	0.38	-	-
PEW (yes vs. no)	-	-	-	-	-	-	1.835 (1.297–2.596)	0.001

DPI, dietary protein intake; PEW, protein energy wasting; HR, hazard ratio; CI, confidence interval; No., numbers. HR and 95% CI were analyzed using multivariate Cox proportional hazard regression analysis. The common covariates of models 3–6 were age, sex, current smoking, alcohol drinking, hypertension, diabetes, causes of chronic kidney disease, urine protein to creatinine ratio, blood urea nitrogen, estimated glomerular filtration rate, bilirubin, hemoglobin, and high sensitivity C-reactive protein.

## Supplementary Materials

The following are available online at http://www.mdpi.com/2072-6643/11/1/121/s1, Table S1: Sensitivity analysis for the effect of DPI on renal survival. Table S2: Association between DPI and PEW components. Figure S1: Development of renal events according to the stage of chronic kidney disease.
