# Supplementary material for: Dietary Protein Intake, Protein Energy Wasting, and the Progression of Chronic Kidney Disease: Analysis from the KNOW-CKD Study
Source: Nutrients. 2019 Jan 8;11(1):121. doi: 10.3390/nu11010121 (PMC6356719; doi:10.3390/nu11010121)

Supplementary materials

Table S1. Sensitivity analysis for the effect of DPI on renal survival

	Model 7		Model 8	
	HR (95% CI)	P	HR (95% CI)	P
DPI tertile				
Second tertile vs. first tertile	0.736 (0.552–0.983)	0.04	0.798 (0.590–1.081)	0.14
Third tertile vs. first tertile	0.693 (0.498–0.963)	0.03	0.773 (0.537–1.114)	0.17
BMI (kg/m^2)	-	-	0.989 (0.941–1.04)	0.68
Estimated SMM (kg)	-	-	0.987 (0.957–1.018)	0.41
Cholesterol < 3.8 mmol/l (yes vs. no)	-	-	1.027 (0.769–1.371)	0.86
Serum albumin < 40.0 g/l (yes vs. no)	-	-	1.401 (1.016–1.931)	0.04

DPI, dietary protein intake; HR, hazard ratio; CI, confidence interval; BMI, body mass index; SMM, skeletal muscle mass. HR and 95% CI were analyzed using Cox proportional hazard regression analysis. In multivariate analysis, covariates in model 1 were age, sex, current smoking, alcohol drinking, systolic and diastolic blood pressure, administration of renin-angiotensin-aldosterone inhibitors, diuretics, beta blocker, calcium channel blockers, and diuretics, fasting glucose, administration of insulin and oral-antidiabetic drugs, causes of chronic kidney disease, urine protein to creatinine ratio, blood urea nitogren, estimated glomerular filtration rate, bilirubin, hemoglobin, and high sensitivity C-reactive protein. Covariates in model 2 were variables in model 1 with BMI, estimated SMM, cholesterol, and serum albumin.

Table S2. Association between DPI and PEW components

	Per 1 g/kg/day increase of DPI			
	Univariate		Multivariate	
	OR (95% CI)	*P*	OR (95% CI)	*P*
Low BMI	0.134 (0.085–0.213)	<0.001	0.482 (0.275–0.845)	0.01
Low estimated SMM	0.052 (0.031–0.087)	<0.001	0.077 (0.041–0.143)	<0.001
Low serum albumin	0.703 (0.491–1.007)	0.06	1.176 (0.699–1.979)	0.54
Low cholesterol	0.890 (0.657–1.207)	0.45	0.973 (0.653–1.451)	0.90

DPI, dietary protein intake; PEW, protein energy wasting; BMI, body mass index; SMM, skeletal muscle mass; OR, odds ratio; CI, confidence interval. DPI was independent variable and four PEW components were dependent variables. OR and 95% CI were calculated using logistic regression analysis. In multivariate analysis, covariates were age, sex, current smoking, alcohol drinking, hypertension, diabetes, causes of chronic kidney disease, urine protein to creatinine ratio, blood urea nitrogen, estimated glomerular filtration rate, bilirubin, hemoglobin, high sensitivity C-reactive protein, BMI, estimated SMM, serum albumin, and cholesterol. When PEW components were chosen as dependent variables, it was removed from the model.

Figure S1. Development of renal events according to the stage of chronic kidney disease. Cr, creatinine; eGFR, estimated glomerular filtration rate.

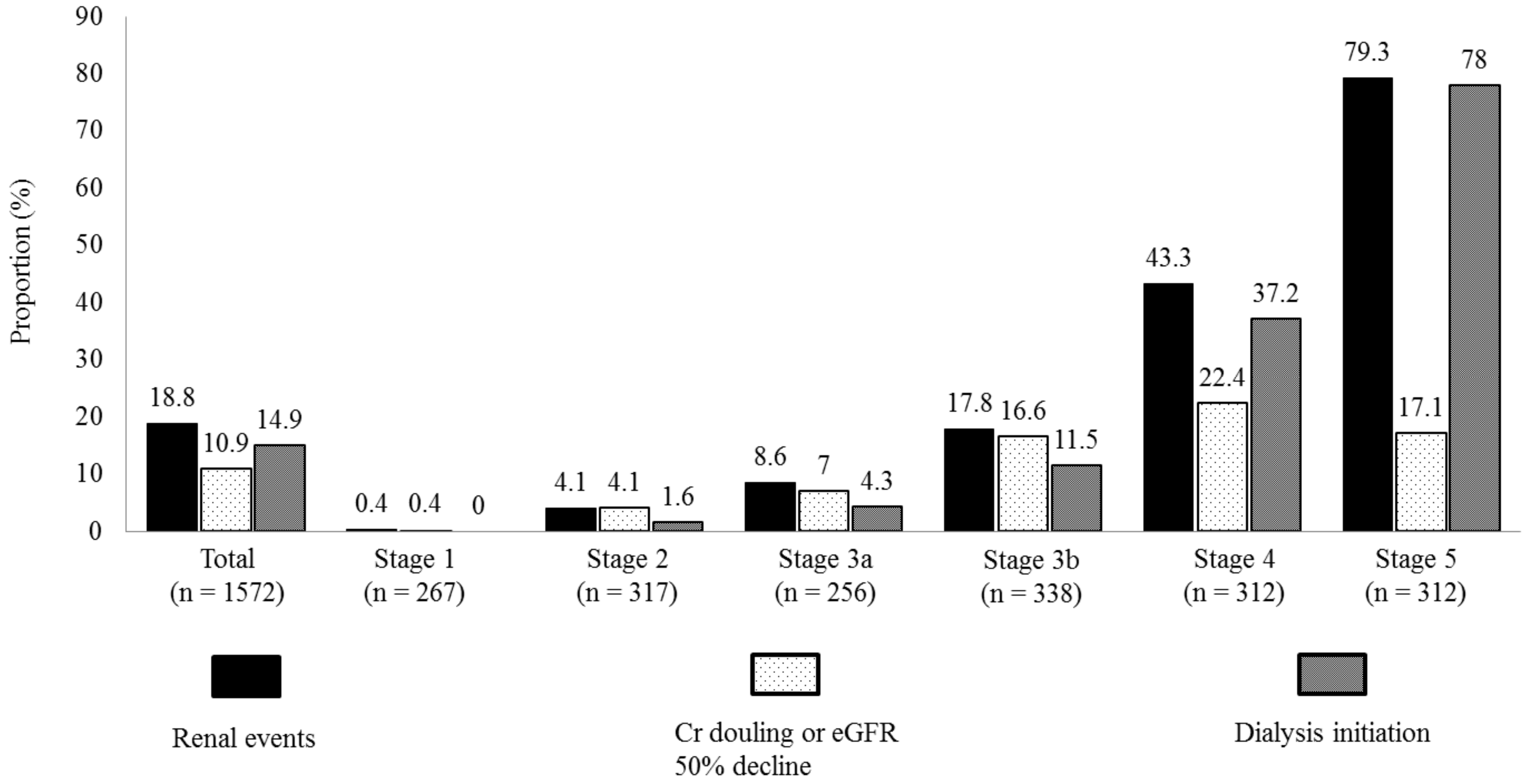

Supplement: Supplementary file 1 [file nutrients-11-00121-s001.pdf]
